# Introducing directly induced microglia-like (iMG) cells from fresh human monocytes: a novel translational research tool for psychiatric disorders

**DOI:** 10.3389/fncel.2015.00184

**Published:** 2015-05-27

**Authors:** Masahiro Ohgidani, Takahiro A. Kato, Shigenobu Kanba

**Affiliations:** ^1^Department of Neuropsychiatry, Graduate School of Medical Sciences, Kyushu UniversityFukuoka, Japan; ^2^Brain Research Unit, Innovation Center for Medical Redox Navigation, Kyushu UniversityFukuoka, Japan

**Keywords:** microglia, regenerative medicine, translational research, psychiatric disorders, schizophrenia, mood disorders, GM-CSF, IL-34

## Abstract

Microglia, glial cells with immunological functions, have been implicated in various neurological diseases and psychiatric disorders in rodent studies, and human postmortem and PET studies. However, the deeper molecular implications of living human microglia have not been clarified. Here, we introduce a novel translational research approach focusing on human microglia. We have recently developed a new technique for creating induced microglia-like (iMG) cells from human peripheral blood. Two cytokines, GM-CSF and IL-34, converted human monocytes into the iMG cells within 14 days, which show various microglial characterizations; expressing markers, forming a ramified morphology, and phagocytic activity with various cytokine releases. We have already confirmed the applicability of this technique by analyzing iMG cells from a patient of Nasu-Hakola disease (NHD; Ohgidani et al., 2014). We herein show possible applications of the iMG cells in translational research. We believe that this iMG technique will open the door to explore various unknown dynamic aspects of human microglia in psychiatric disorders. This also opens new routes for psychopharmacological approach such as drug efficacy screening and personalized medicine.

Recently, the roles of microglia, immune cells in the brain, have been highlighted not only by neuroscientists but also by a variety of clinical researchers, especially in the field of neurology and psychiatry (Hughes, 2012). The pathophysiology of microglia has been suggested in various neuronal diseases and psychiatric disorders by human postmortem and positron emission tomography (PET) studies (Steiner et al., 2008; van Berckel et al., 2008; Takano et al., 2010; Gupta et al., 2014). Furthermore, microglial modulation has been proposed as an intervention in brain diseases including psychiatric disorders by recent clinical trials using minocycline, an antibiotic with microglial inhibitory effects (Miyaoka et al., 2008; Levkovitz et al., 2010; Chaudhry et al., 2012; Hayakawa et al., 2014). It has also been suggested that minocycline acts to modulate social interactions not only in psychiatric patients but also in healthy volunteers (Kato et al., 2012; Watabe et al., 2012, 2013). On the other hand, various psychotropic drugs have been revealed to inhibit microglial over-activation in *in vitro* experiments using rodent cells (Kato et al., 2007, 2008, 2011a,b; Bian et al., 2008; Seki et al., 2013).

The above reports have strongly suggested that maladaptive microglial activation may play a crucial role in various brain disorders (Block et al., 2007; Hanisch and Kettenmann, 2007; Kato et al., 2013). However, the deeper dynamic molecular actions of living human microglia in real patients have not been well clarified due to technical and ethical issues (such as difficulties involved in human brain biopsies). Until now, almost all human microglia research has been conducted using the postmortem brain or PET. These approaches have revealed important roles for activated microglia in psychiatric patients such as schizophrenia and autism (Steiner et al., 2008; van Berckel et al., 2008; Takano et al., 2010; Suzuki et al., 2013; Gupta et al., 2014). However, using these methods, it is difficult to ascertain the molecular pathological mechanisms such as the signaling pathway and gene expression pattern. On the other hand, animal (rodent) studies with cytological and histological analysis can reveal the various deeper dynamic actions of microglia at molecular levels (Nimmerjahn et al., 2005; Wake et al., 2009; Kettenmann et al., 2011). In fact, microglial activation has been shown in a variety of cytological and histological analyses using rodent models of brain diseases (Wu et al., 2002; Yoshiyama et al., 2007). Molecular biological analysis using rodent primary cultures and cell strains has been the standard method until now (Kato et al., 2007, 2008, 2011a,b; Bian et al., 2008; Seki et al., 2013; Mizoguchi et al., 2014a,b). In fact, rodent studies have been very vital in microglial research, however the question remains- how much does human pathology reflect in rodents? Can schizophrenia model mice have delusions and/or hallucinations? Even though a variety of rodent models of psychiatric disorders exist, it is extremely hard to validate deeper psychopathologies in rodents.

Thus, human studies using living brain cells derived from psychiatric patients have been warranted to evaluate the interactions of microglial activities and deeper psychopathology including psychiatric symptoms. A novel term “cellular model” has emerged in addition to “animal model” in the research of various physical diseases. The technology to obtain human neuronal cells from non-brain tissues (e.g., skin) by novel reproductive techniques such as induced pluripotent stem (iPS) cells (Takahashi et al., 2007) and directly induced neuronal (iN) cells (Pang et al., 2011; Liu et al., 2013) has emerged, and these tools have just recently been applied to psychiatric research (Kim, 2010; Brennand et al., 2011; Wen et al., 2014). Further, iPS technology can produce glial cells such as astrocytes (Juopperi et al., 2012) and progenitor of oligodendrocytes (Wang et al., 2013). However, to our knowledge, there exist no reports on the production of microglia by iPS technology. Very recently, we have developed a novel technique to create induced microglia-like cells (iMG) from human peripheral blood (Ohgidani et al., 2014).

The brain is mostly composed of ectomorphic cells such as neurons, astrocytes and oligodendrocytes. Microglia is the only mesomorphic cell in the brain (Kettenmann et al., 2011). Therefore, we tried to induce microglia-like cells from peripheral monocytes, which have the same origin as mesomorphic cells. To determine what cytokines can induce iMG cells from human peripheral monocytes, we tested the effects of cytokines. Surprisingly, the cocktail of both granulocyte-macrophage colony-stimulating factor (GM-CSF) and interleukin (IL) −34 successfully induced small soma bodies bearing numerous branched collaterals, which expressed the specific morphology of ramified microglia-small soma with extensive radial ramifications. The iMG cells express the essential characteristics of human microglia such as surface markers and drug responses, phagocytosis and cytokine production (Figure 1).

**Figure 1:**
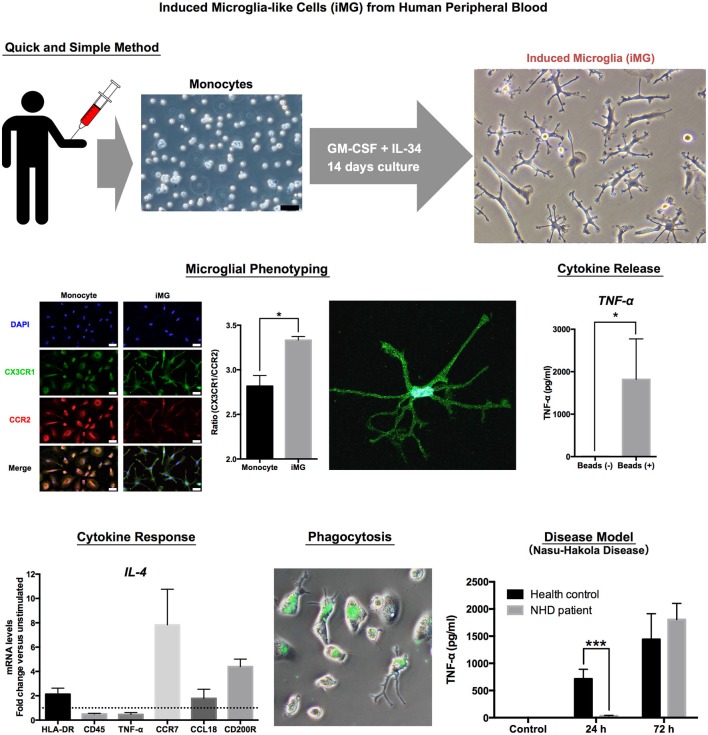
**Induced Microglia-like Cells (iMG) from Human Peripheral Blood**.

We first utilized the iMG cells as a “cellular disease model” focusing on one of the most famous primary microglia-oriented diseases, the Nasu-Hakola disease (NHD; Hakola, 1972; Nasu et al., 1973). NHD is a very rare autosomal recessive disease, and the responsible two genes, which are expressed in microglia in the brain, are DNAX-activation protein of 12 kDA (DAP12) and triggering receptor expressed on myeloid cells 2 (TREM2; Paloneva et al., 2000, 2002). The deeper pathophysiological roles of microglia have not been well understood. Thus, we investigated the pathophysiology of NHD using the iMG technique. In agreement with genetic diagnosis, the iMG cells from a NHD patient (141delG in DAP12 gene) showed significantly lower expression of DAP12 than those from a healthy control (HC). Interestingly, the response of producing pro-inflammatory cytokines (TNF-α and IL-6) was delayed in the iMG cells from the NHD patient as compared to those from HC. Furthermore, we have also confirmed the delayed cytokine productions in the NHD model of iMG cells which was prepared by siRNA (Ohgidani et al., 2014). These novel findings may help to understand the hitherto unknown pathophysiological aspects of NHD.

In this way, we believe that the iMG technique will enable the clarification of novel pathophysiological dysfunctions of human microglia as a translational research tool in various brain diseases including psychiatric disorders. We believe that the iMG technique will enable the exploration and development of psychiatric research especially to the following areas;

## Multidimensional Correlation Analysis with Clinical Data, Brain Imaging Data and iMG

**Figure 2:**
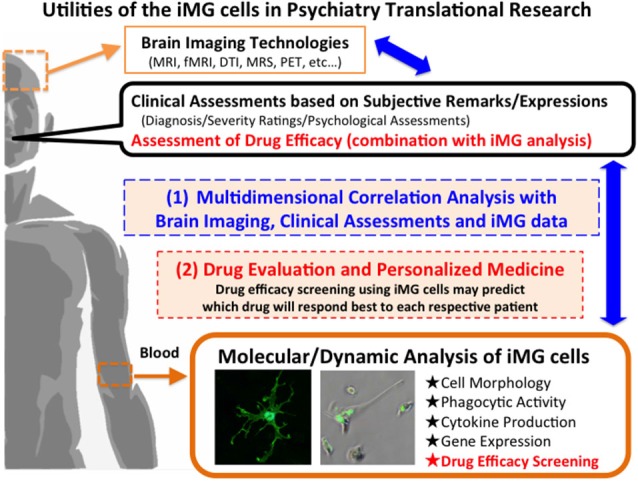
**Utilities of the iMG cells in Psychiatry Translational Research**.

By combining clinical data, brain imaging data, and the iMG data from the same patient will be able to clarify the dynamic interaction between a specific psychopathology and a specific microglial activation (Figure 2-(1)). For example, the aberration of TREM, which is expressed in microglia, has recently been observed in psychiatric disorders such as bipolar disorder (Weigelt et al., 2011) and Alzheimer’s disease (Jonsson et al., 2013). Analyzing the TREM aberration of iMG cells from psychiatric patients can help to clarify the main role of TREM in psychopathology, which in turn may assist in the psychiatric evaluation of diagnosis and severity.

## Drug Evaluation and Personalized Medicine

We previously reported the neuroprotective effects of psychotropic drugs via suppressing microglial activation using rodent microglial cells (Kato et al., 2007, 2008, 2011a,b; Bian et al., 2008; Seki et al., 2013). The iMG technique may help to predict drug responses before treating patients. Drug efficacy screening using iMG cells can predict which drug will respond best to each respective patient, and the technique may be applied as a companion diagnostic tool, which has raised expectations for the application of “order-made” medicine with a reduction in side effects and a shortening of treatment period (Figure 2-(2)).

## Conclusion

We introduced a novel translational research approach focusing on human microglia-like cells using the iMG technique. Before reaching a conclusion, we need to mention a recent considerable discussion regarding functional differences between yolk sac derived microglia and monocyte derived microglia (Ginhoux et al., 2010; Katsumoto et al., 2014). We should keep it in our mind that the iMG cells are originated from monocytes, which may be different from the functions of yolk sac derived microglia. In addition, because IL-34 is a tissue-restricted ligand of CSF1R and this cytokine is associated with the development of other types of monocyte-derived cells such as Langerhans cells and possibly dendritic cells (Wang et al., 2012), which should also be considered. Despite these propositions, we believe that the iMG technique, at least to some extent, can analyze human microglial pathology in a living state, which had been impossible until recently. We hope that this translational method will open the door to explore various unknown dynamic aspects of human microglia in brain diseases, especially psychiatric disorders. This opens new routes for not only understanding the psychopathological mechanism of psychiatric disorders but also psychopharmacological approach such as drug efficacy screening and personalized medicine.

## Conflict of Interest Statement

The authors declare that the research was conducted in the absence of any commercial or financial relationships that could be construed as a potential conflict of interest.
